# Critical Velocity, Maximal Lactate Steady State, and Muscle MCT1 and MCT4 after Exhaustive Running in Mice

**DOI:** 10.3390/ijms242115753

**Published:** 2023-10-30

**Authors:** Juan B. Orsi, Lara S. Araujo, Pedro P. M. Scariot, Emanuel E. C. Polisel, Luisa O. Cardoso, Claudio A. Gobatto, Fúlvia B. Manchado-Gobatto

**Affiliations:** Laboratory of Applied Sport Physiology, School of Applied Sciences, University of Campinas, Pedro Zaccaria Street, 1.300, Jardim Santa Luíza, Limeira 13484-350, São Paulo, Brazil; larasoaresdearaujo@hotmail.com (L.S.A.); pedroppms@yahoo.com.br (P.P.M.S.); emanuel_camolese@hotmail.com (E.E.C.P.); cardosoluisa97@gmail.com (L.O.C.); cgobatto@unicamp.br (C.A.G.); fgobatto@unicamp.br (F.B.M.-G.)

**Keywords:** blood lactate, monocarboxylate transporters, aerobic capacity, running mice, mathematical models, physiological parameters

## Abstract

Although the critical velocity (CV) protocol has been used to determine the aerobic capacity in rodents, there is a lack of studies that compare CV with maximal lactate steady state intensity (iMLSS) in mice. As a consequence, their physiological and molecular responses after exercise until exhaustion at CV intensity remain unclear. Thus, we aimed to compare and correlate CV with iMLSS in running mice, following different mathematical models for CV estimation. We also evaluated their physiological responses and muscle MCT1 and MCT4 after running until exhaustion at CV. Thirty C57BL/6J mice were divided into two groups (exercised-E and control-C). Group E was submitted to a CV protocol (4 days), using linear (*lin1* and *lin2*) and hyperbolic (*hyp*) mathematical models to determine the distance, velocity, and time to exhaustion (tlim) of each predictive CV trial, followed by an MLSS protocol. After a running effort until exhaustion at CV intensity, the mice were immediately euthanized, while group C was euthanized at rest. No differences were observed between iMLSS (21.1 ± 1.1 m.min^−1^) and CV estimated by *lin1* (21.0 ± 0.9 m.min^−1^, *p* = 0.415), *lin2* (21.3 ± 0.9 m.min^−1^, *p* = 0.209), and *hyp* (20.6 ± 0.9 m.min^−1^, *p* = 0.914). According to the results, CV was significantly correlated with iMLSS. After running until exhaustion at CV (tlim = 28.4 ± 8,29 min), group E showed lower concentrations of hepatic and gluteal glycogen than group C, but no difference in the content of MCT1 (*p* = 0.933) and MCT4 (*p* = 0.123) in soleus muscle. Significant correlations were not found between MCT1 and MCT4 and tlim at CV intensity. Our results reinforce that CV is a valid and non-invasive protocol to estimate the maximal aerobic capacity in mice and that the content of MCT1 and MCT4 was not decisive in determining the tlim at CV, at least when measured immediately after the running effort.

## 1. Introduction

The scientific community has widely used laboratory rodents in investigations on the acute and chronic effects of both disease prevention and physical performance [1,2]. Despite some concerns about the extrapolation of knowledge, animal models have practical advantages over research on human beings, such as easy manipulation, greater control of intrinsic and extrinsic variables, and the possibility of more invasive procedures [3]. It is also worth highlighting that rodents show physiological responses very near to those of humans subjected to physical exercise, as observed by Goutianos et al. [4] on blood parameter responses and Veskoukis et al. [5] on basic blood redox/inflammatory profiles. 

Due to the high number of studies with exercised rodents, proper exposure to exercise should involve precise control and good practices, requiring protocols that can evaluate their physical capacities with maximum precision and reliability [6,7,8]. Research has increasingly proposed the use of adapted protocols originally developed for humans in animal models. In this sense, studies with exercised rodents have focused on the development of protocols to determine the intensity of transition between the predominance of aerobic and anaerobic metabolisms in energy supply. Among them, the maximal lactate steady state (MLSS) stands out as the gold standard for assessing aerobic capacity [9,10,11]. The MLSS intensity (iMLSS) is equivalent to the maximal exercise effort in which the production and removal of blood lactate remain in balance. The MLSS protocol is comprised of three or four continuous efforts with blood collections to determine the lactatemia curve throughout the tests [12,13,14,15]. Thus, efforts maintained at intensities equal to or lower than the iMLSS would avoid the accumulation of blood lactate [16]. Nevertheless, the determination of iMLSS requires tests with several constant loads across several days and frequent blood collections, making this an invasive method [17].

The use of protocols to assess aerobic capacity based on practical, non-invasive, and low-cost tests is, therefore, highly desirable and attractive. In this context, the critical velocity protocol (CV) has proved to be an interesting alternative since it can estimate aerobic capacity without blood collection [18]. Derived from the intensity versus time relationship for a series of three to four exhaustive efforts (predictive trials), the CV represents the exercise intensity that can be maintained by aerobic metabolism, as it demarcates the transition between heavy and severe domains [19,20,21,22]. The protocol also provides another parameter, namely, anaerobic running capacity (ARC), which, although less explored, seems to be an anaerobic indicator [23,24]. 

Previous studies have shown the viability of the CV protocol in several mouse [25,26,27] and rat (Wistar and Sprague Dawley) [11,25,28,29] strains subjected to treadmill running. Such a protocol has also been successfully prescribed for aerobic physical training in C57BL/6J runners [30,31]. However, although reproducible and already compared with the anaerobic threshold in this mouse strain, to the best of our knowledge, CV has not yet been compared and correlated with iMLSS in mice. 

The literature has scarce information on how C57BL/6J runners maintain efforts at CV. Using Sprague Dawley rats, Copp et al. [28] showed that, at constant intensities below ~15% of the CV, mice can maintain efforts and stable oxygen uptake (VO_2_) for long periods (>40 min), meeting CV theoretical assumptions [18,21,32]. Copp et al. [28] also found greater recruitment of glycolytic muscle fibers and a considerable increase in VO_2_ with rapid exhaustion (~10 min) at intensities ~15% above the CV. Given the relevance of energy aspects and blood lactate balance to maintain an effort at a maximal aerobic intensity [33,34], investigations with rodents can better explore the physiological and molecular aspects of CV. With the exception of Copp et al. [28], the literature has no data describing the profile of physiological responses in running rodents due to efforts at CV intensity. 

Physical exercise is understood to be an important modulator of molecular signaling pathways. There is evidence that just one session of physical exercise is capable of triggering a series of cellular responses that result in the transcription and translation of proteins [9]. Protein changes at the cellular level certainly contribute to physiological and physical performance adaptations [35,36,37,38]. It is known that proteins related to lactate transport (monocarboxylate transporters—MCTs) are highly sensitive to exposure to physical exercise [39]. Corresponding to this, Araujo et al. [9] observed an increase in the gene expression of MCTs in the oxidative (soleus) muscle immediately after a single swimming session performed at an aerobic intensity equivalent to the MLSS, corroborating the literature, which indicates the relevant contribution of MCTs to the acid-base balance [40,41]. Given the above, we strongly believe that the protein content of MCTs in skeletal muscles is related to the physiological stability of the organism, which is necessary for the maintenance of continuous efforts at intensities equivalent to the maximal aerobic capacity and the critical velocity reached during exercise until physical exhaustion (time to exhaustion—tlim), having an important inter-individual variation. 

Therefore, the present study sought to compare and correlate CV with iMLSS in running rats. Considering that CV and ARC estimates may differ according to the mathematical model adopted, this study also aimed to compare the CV, ARC, and R^2^ values obtained by three mathematical models (hyperbolic and linear 1 and 2), as well as some physiological responses and muscle protein content of mice after a running effort at an individual CV intensity until exhaustion and at rest (group C). Finally, we investigated the correlations between physiological and molecular parameters and time to exhaustion at CV intensity. We specifically analyzed serum parameters (glucose, cholesterol, triglycerides, albumin, and urea) and tissue energy stores (glycogen). In a molecular way, we determined the protein content of isoforms 1 and 4 of monocarboxylate transporters (MCTs) involved in blood lactate balance [42,43]. We hypothesized that (i) the CV obtained by different mathematical models would be equal to and highly correlated with iMLSS; (ii) there would be no differences between CV, ARC, and R^2^ estimated by the hyperbolic and linear models; (iii) the physiological responses observed in group E after exhaustive running at CV intensity would be different from those of control animals; and (iv) a significant correlation would be found between time to exhaustion at CV intensity and physiological responses and muscle MCT1 and MCT4. In the latter case, considering that energy metabolism is strongly involved in exercise tolerance, we expected to observe a greater depletion of glycogen stores and higher levels of MCT1 and MCT4 proteins in mice capable of maintaining longer exercise efforts at CV intensity.

## 2. Results

### 2.1. CV Protocol and Mathematical Models

**Figure 1:**
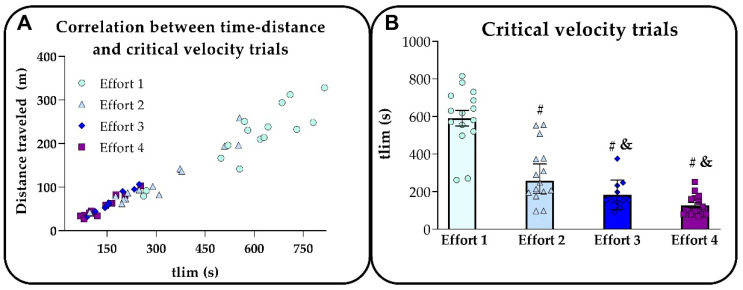
(**A**) illustrates the relationship between tlim and distance traveled in the four predictive tests (critical velocity trials) at different exercise intensities (m.min^−1^), while (**B**) shows the difference in tlim. The average intensities of efforts 1, 2, 3, and 4 were 21.6 ± 0.9, 22.7 ± 0.8, 23.7 ± 0.5, and 24.5 ± 0.9 m.min^−1^, respectively. It should be mentioned that the exercise intensities were chosen individually for each rodent so that tlim would neither exceed 15 min nor remain below 1 min. Statistical analysis: # different from effort 1; & different from effort 2.

We plotted tlim versus distance traveled for all mice in the four exhaustive running tests (Figure 1A) to illustrate the great consistency of our data. Figure 1B shows a lower tlim at higher intensities (efforts 2, 3, and 4) than at lower ones (effort 1). In addition, efforts 3 and 4 had a lower tlim than effort 2.

Table 1 shows the ARC and R^2^ values obtained by the three mathematical models used (*lin1*, *lin2*, and *hyp*). No significant differences were found between *lin1* and *lin2* (*p* = 0.143, ES = 0.08). The CV *hyp* was significantly lower than that in *lin1* (*p* = 0.013, ES = 0.14) and *lin2* (*p* < 0.001, ES = 0.21). In the *hyp* model, the ARC was significantly higher than in *lin1* (*p* = 0.035, ES = 0.50) and *lin2* (*p* = 0.008, ES = 0.79). No differences were observed in ARC values between *lin1* and *lin2* (*p* = 0.323, ES = 0.31). *Lin1* showed better coefficients of determination (R^2^ = 0.99 ± 0.00).

### 2.2. iMLSS Results 

**Figure 2:**
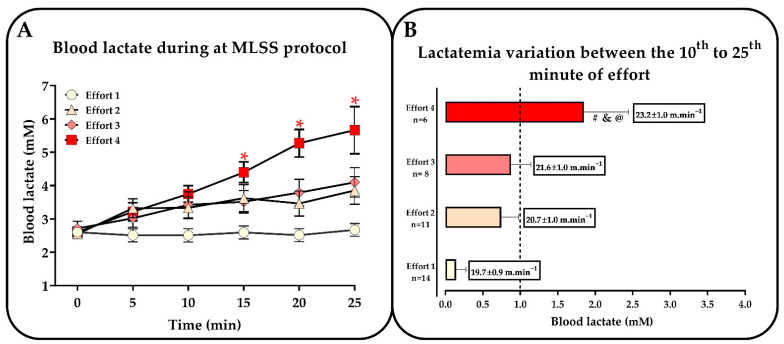
(**A**) Blood lactate concentration curve during several MLSS tests and (**B**) variation in blood lactate (∆) between the 10th and 25th minute of effort. Note that the MLSS intensity is reached in the third effort (the highest exercise intensity), causing a blood lactate variation of less than 1 mM between the 10th and 25th of exercise. ANOVA referring to lactate analyses throughout the MLSS tests (**A**): * different from blood lactate at rest (0 min). Blood lactate variation (**B**): # different from effort 1; & different from effort 2; @ different from effort 3.

As illustrated in Figure 2, the mice showed a clear stabilization of blood lactate concentration (which varied less than 1 mM between the 10th and 25th minute of effort) during efforts 1 (19.7 ± 0.94 m.min^−1^), 2 (20.7 ± 1.00 m.min^−1^), and 3 (21.6 ± 1.01 m.min^−1^). However, in effort 4 (23.2 ± 1.03 m.min^−1^), the blood lactate concentration changes exceeded 1 mM between the 10th and 25th minute (Figure 2). The increment in blood lactate at the end of exercise (in relation to rest) became more evident as the intensity increased (e.g., in effort 4). An individual iMLSS of 21.1 ± 1.10 m.min^−1^ was observed at an MLSS concentration of 3.4 ± 1.2 mM.

### 2.3. Comparisons and Correlations between CV and iMLSS

**Figure 3:**
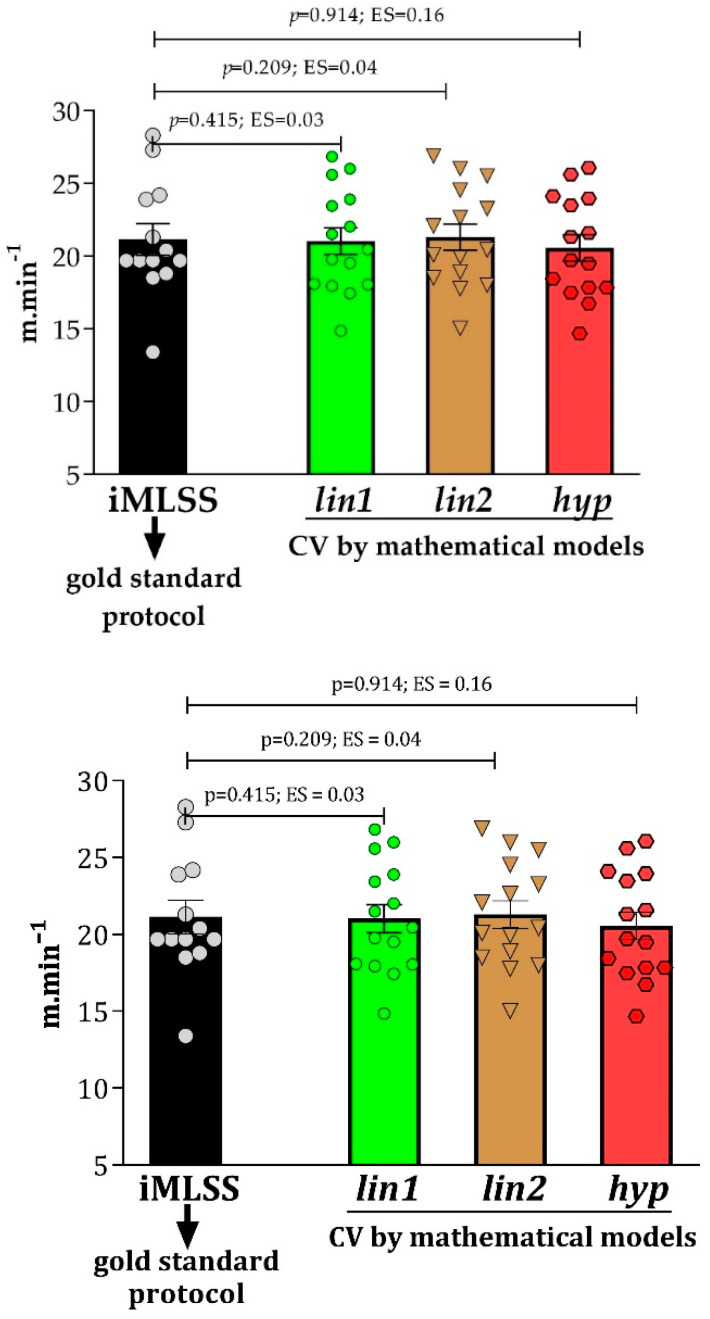
Comparison between iMLSS and CV estimated from three mathematical models (*lin1*, *lin2*, and *hyp*).

Figure 3 shows that no statistical differences were found between iMLSS and CV in the three mathematical models used, resulting in a small ES (ES ≤ 0.49). 

**Figure 4:**
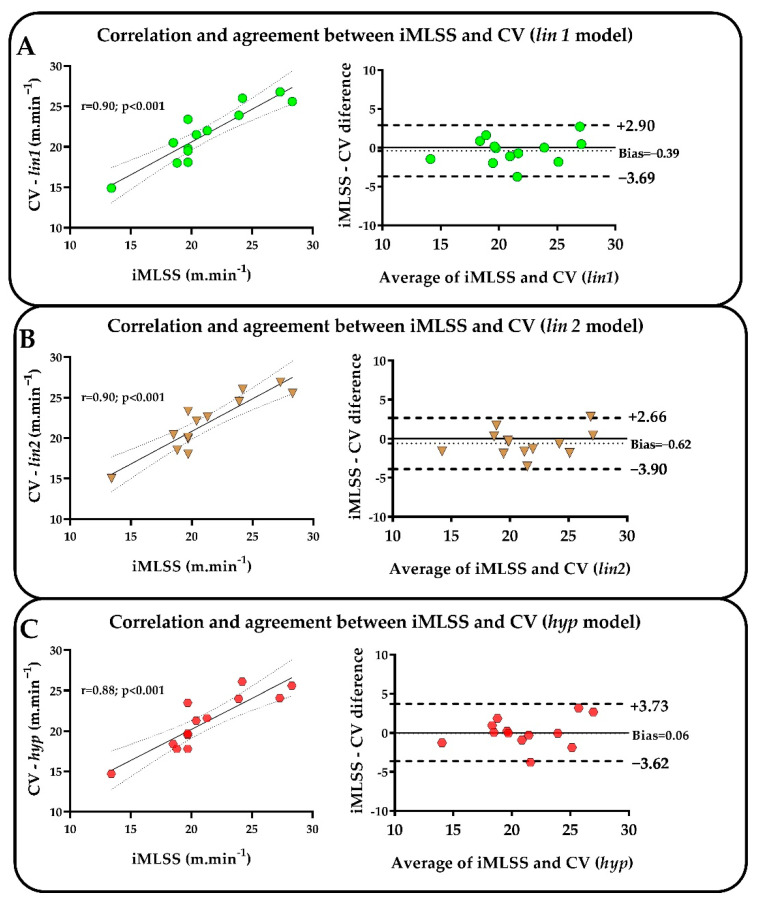
Correlation and agreement analysis represented by the Pearson correlation (at the left) and Bland–Altman graphs (at the right), respectively. iMLSS values were tested against the estimated CV values from three mathematical models, lin1 (green circles, (**A**)), lin2 (brown triangles, (**B**)), and hyp (red hexagons, (**C**)).

Pearson’s product–moment revealed a significant correlation between iMLSS and CV obtained by the three mathematical models employed, while Bland–Altman analysis showed good agreement between these parameters (Figure 4A–C).

### 2.4. Biochemical and Biomolecular Responses to Exhaustive Efforts at CV Intensity

According to our comparative analysis (independent samples *t*-test), group E showed lower urea values than group C (Table 2). Regarding the serum parameters (glucose, cholesterol, triacylglycerol, and albumin), no differences were observed between these groups. However, group E showed lower skeletal liver and muscle (gluteus maximus) tissue glycogen stores (Table 2) than the control group. No differences were found in other tissues between the groups. 

**Figure 5:**
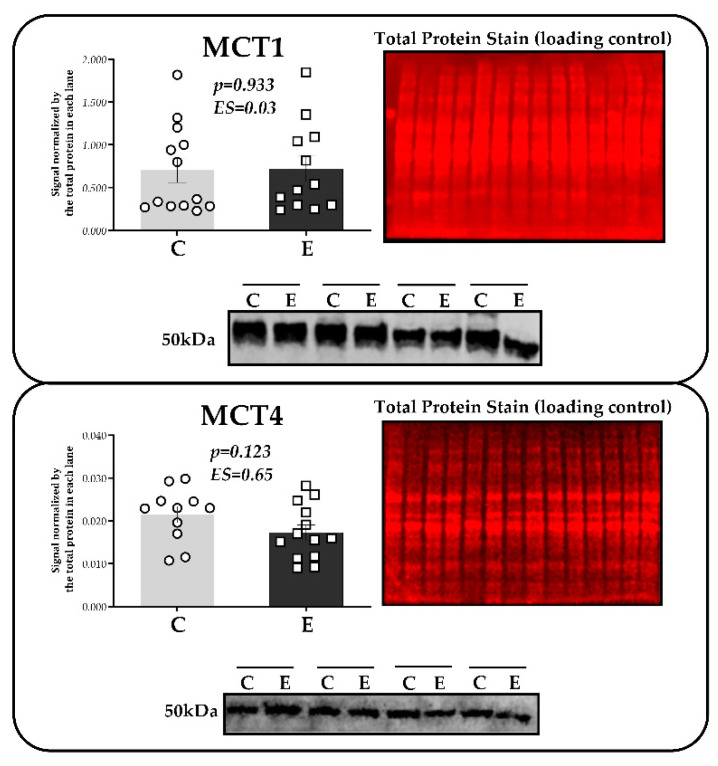
MCT1 and MCT4 protein content in the soleus muscle of animals from the C (rest) and E (after exhaustive efforts at CV intensity) groups. The figure illustrates a representative Western blot using MCT1 antibody detection and a membrane after staining with total Protein REVERT staining^®^. All membranes were stained for total protein to normalize interfacial differences in protein loading. The bars on the charts represent means and standard errors (n = 11–13 per group).

No differences were observed in the MCT1 and MCT4 protein content present in the soleus muscles between the exercised and control groups, as illustrated in Figure 5.

**Figure 6:**
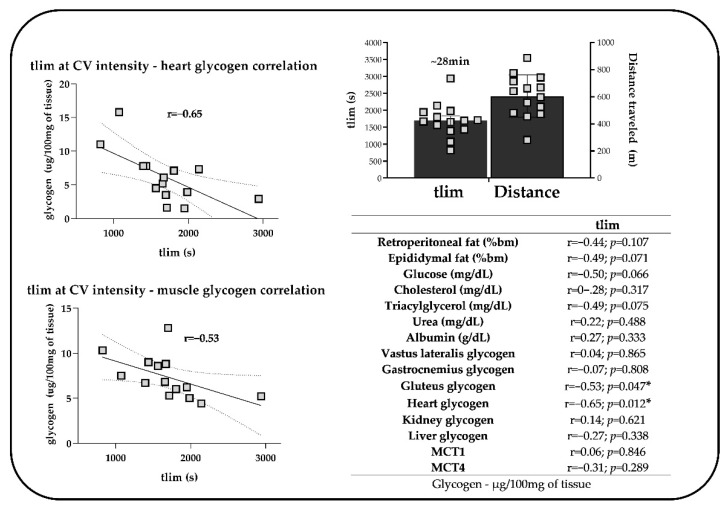
Correlations between tlim and physiological parameters at CV intensity in the exercised group. * Significant correlation between physiological parameters and tlim.

Pearson’s test was used to seek significant correlations between physiological responses and tlim at intensities equivalent to CV (Figure 6). According to the results, although there were significant and inverse correlations between tlim and cardiac and gluteal glycogen levels. No significant correlation was found between tlim and other physiological parameters at the end of exhaustive efforts at CV intensity.

## 3. Discussion

Our main findings revealed an equal and significant correlation between the CV obtained by three mathematical models and iMLSS, the gold standard for determining maximal aerobic capacity. Additionally, there was good agreement between the aerobic parameter determined by the MLSS protocol and the CV estimated by a non-invasive protocol. These findings validate CV as a measure of maximal aerobic capacity with greater consistency and reliability. This is undoubtedly the main contribution of this study since it allows the reliable use of CV in different contexts to assess aerobic capacity or prescribe aerobic exercises to a mouse model. Although blood lactate can provide important information about metabolic demand during efforts, such measurements are often inaccessible, making it difficult to adequately prescribe exercise intensities. In this sense, our results reinforce the use of CV in running mice, as long as individual predictive intensities (CV trials) are chosen so that the animals reach exhaustion between 1 and 15 min.

The mean CV values found (~21 m.min^−1^) are in agreement with Billat et al. [25], who observed values around 18 m.min^−1^ in C57BL/6J mice. Previous studies by our group also showed a CV of 16–18 m.min^−1^ for sedentary C57BL/6J mice and ~24 m.min^−1^ for those undergoing aerobic physical training [31]. Other studies reported a CV variation between 22 and 28 m.min^−1^ [25,26,27] for other mouse strains (CD1, FVB/N, BERK-SS, and Friend Virus B-type mice). Similar magnitudes were also found in Wistar rats, with a CV value around 22 m.min^−1^ [11,29]. Regarding Sprague Dawley rats, since they can run at high speeds [44,45], they reach a CV at substantially higher intensities (~48 m.min^−1^) [28]. 

As also observed by Copp et al. [28], a greater R^2^ was obtained in *lin1* (R^2^ = 0.99) than in *lin2* (R^2^ = 0.71) and *hyp* (R^2^ = 0.85). On the other hand, it was possible to observe significant differences between the parameters derived from the mathematical models. More specifically, *hyp* resulted in a lower CV intensity than the other two linear models (a variation of ~1.94% and ~3.40% of variation in relation to the CV values in lin1 and lin2, respectively). In fact, CV estimates require methodological analyses, as they may depend on different mathematical models and effort numbers [46,47,48,49]. Despite that, the CV intensity estimated by the different mathematical models employed did not differ from the iMLSS value, which was 21.1 ± 1.10 m.min^−1^. This not only shows the robustness of the CV protocol but also reinforces its physiological coherence as an estimate of maximal aerobic capacity since the literature considers iMLSS to be the gold standard. With respect to MLSS concentration, the blood lactate value found herein was 3.4 ± 1.2 mM, a value similar to that observed by Gobatto et al. [10] in Swiss mice submitted to swimming exercise.

In addition to its strong relationship with iMLSS, another evidence that reinforces the aerobic profile of CV is the tlim at this intensity (~28 min), a parameter that seems to be adequate for aerobic efforts (at MLSS intensities). Our results agree with Copp et al. [28], who showed that at constant intensities below ~15% of the CV, exercise may last for long periods (>40 min) as mice stabilize VO_2_. If we consider studies in humans, our tlim data at CV agree with the literature, which reports a range between 15 and 30 min at a critical power intensity [50,51,52,53].

The central role of tlim in CV estimates deserves further discussion. Despite the widespread use of “exhaustive” protocols in rodents, the literature still lacks consensus on the optimal criteria to define the endpoint of exercises, given the small number of studies on this issue. However, our methodological care may have contributed to a more consistent criterion. In addition to choosing a strain of mice capable of running well (C57BL/6J), we familiarized our exercise group with treadmill running before evaluating them. The experiments were conducted under the following conditions: (a) no electrical stimuli were used during treadmill running; (b) the warm-ups preceded all tests; and (c) experienced researchers considered the inability of mice to run adequately on the treadmill (despite a light touch with a soft brush) as tlim. Moreover, the experiments were performed at night under a red light (6 p.m. to 9 p.m.), which favors the physical performance of nocturnal rodents following previous observations [54]. These measurements may have contributed to the good fits observed (R^2^), especially in lin1, confirming that the model fits the data well and that consistent and reliable estimates can be made using a non-invasive protocol.

This study also investigated how the demand for aerobic exercise at CV intensity would affect the animals’ physiological responses linked to energy metabolism and blood lactate balance. A significant reduction in liver glycogen (yet with no changes in blood glucose) was observed in the exercised group. This finding is relevant due to the important role played by the liver in maintaining glycemia [55,56]. Our results also showed consistency, as carbohydrates, in the form of glucose and glycogen, constitute an important energy substrate for exercise at intensities close to iMLSS (∼65–80% VO_2_max) [57,58,59]. The maintenance of glycemia at the expense of glycogen breakdown evidences the importance of hepatic glucose production during metabolic challenges in exhaustive efforts (at CV intensity), which may be related to some vital functions such as the preservation of brain function [60]. Despite the great relevance of carbohydrates in the maintenance of effort, aerobic exercises use fatty acids as an energy substrate [57,58,59]. Regarding lipid metabolism during exhaustive CV efforts, no differences were found in triacylglycerols between the exercised and control animals, suggesting that such efforts failed to require a significant increase in this compound in the bloodstream of mice to provide them with energy. We did not expect to find differences in retroperitoneal and epididymal fat between the groups, as we avoided dietary interventions or physical exercise. In this case, we can only use this data to characterize our groups and guarantee similar conditions between them.

A significantly lower amount of glycogen was found in the gluteal maximum tissue of mice exercised at CV, along with significant and inverse correlations between time to exhaustion at CV intensity and cardiac and gluteal maximum glycogen levels, indicating that the tlim increased in rodents that used more glycogen. In line with our observations, studies have reported decreased glycogen in rats, especially in the glutes of those that swam at an individual anaerobic threshold intensity until exhaustion [61]. Thus, it can be speculated that the gluteal maximum in rodents is highly relevant for the maintenance of aerobic efforts, even if motor tasks differ. Moreover, the correlation between cardiac glycogen concentrations and tlim suggests that animals with greater tlim use more cardiac glycogen. Greater cardiac work in the final seconds of exhaustion may demand more glycogen as an energy source. It is worth mentioning that mice have a much higher heart rate than humans, with an average of 250 bpm at rest [62,63], remaining at around 550 bpm during exercises [64] and possibly reaching 650 bpm during exhaustion [65,66]. Therefore, it can be inferred that the increase in cardiac rates to meet energy demand can also trigger exhaustion in running mice—a line of investigation that requires greater attention in future investigations.

Urea results also require discussion due to its strong involvement in protein metabolism [67]. Exercises, especially longer sessions, catabolize proteins, forming urea as a residual product [68,69]. Although an increase in urea is expected after aerobic efforts (since mice already show energy impairments such as lower glycogen), in this work, we observed lower urea concentrations in the exercised group than in the control. This indicates that the protein catabolism did not occur, as reported by Beck et al. [61], who found no serum protein differences between control animals and those subjected to tlim at the swimming anaerobic threshold intensity. Even though further analyses should be conducted to precisely describe how urea decreased in exercised rodents, we can speculate that increased blood flow (due to exercise) accelerates serum urea clearance [70].

We also measured monocarboxylate transporters (MCTs), which act in lactate and hydrogen ion extrusion and uptake [43,71]. The literature on exercise physiology has demonstrated great interest in evaluating MCTs [40,72]. According to evidence, MCT1 and MCT4 are the main isoforms in skeletal muscle tissues. Previous studies established that glycolytic fibers express MCT4, which helps release lactate into extracellular spaces [73], while MCT1, abundant in oxidative fibers, performs the influx of lactate, mainly because of its high affinity with it [74,75,76]. Despite the fact that muscle contractile activities (in several experimental models) can stimulate MCT gene and protein expression [31,76,77,78,79,80,81], the literature has little information on the effects of a single aerobic effort on MCTs.

We hypothesized that the content of MCT protein in the soleus muscle would be higher for mice subjected to exhaustive exercise at CV intensity than for control animals (without exercise stimulus). This hypothesis seemed coherent since Araujo et al. [9] reported an increased MCT1 and MCT4 gene expression in the soleus muscle of mice shortly after exercise (25 min) at iMLSS, even though the increase in mRNA does not necessarily imply protein translation [82,83]. This study found no difference in MCT1 and MCT4 content between the groups. To explain this result, we must consider the biomolecular stimuli of exercise. In terms of tissue collection, although exercise duration and post-exertion duration affect protein expression, we chose to euthanize animals immediately after tlim at CV. Different MCT responses (including greater protein expression) could have been observed if longer intervals between exhaustive effort and tissue extraction were chosen. This is in agreement with Coles et al. [84], who conducted an experiment with Sprague Dawley rats submitted to running exercise at a moderate intensity (21 m.min^−1^, 15% slope). Such protocol consisted of 30 min of exercise and 30 min of rest until completing 2 h of exercise, which in percentage is equivalent to 428.5% of the time performed by mice in our study (~28 min). Although this long period was able to increase MCT4 protein content in the soleus of mice after a single exercise [84], we believe that our experimental design can be used to investigate the elements that contribute to aerobic tolerance at CV intensities. Therefore, additional studies should evaluate protein expression at longer times after exposure to exercise, including the time course of these proteins after exhaustive running effort.

Considering that the literature has already indicated that better performance (aerobic tolerance) is associated with MCTs [85,86,87], we expected to observe a positive and significant correlation between MCT1 and MCT4 and tlim at CV intensity, but we found none. Even though we lack data to support this finding, we speculate that such associations can be more easily found in trained individuals due to their improved metabolic acidosis control. Based only on our data, the content MCTs, at least when measured immediately after an exercise effort at CV intensity in the soleus muscle, had no significant correlation with greater tlim during maximal aerobic effort, suggesting that energy aspects (e.g., gluteal glycogen) are better for determining the aerobic tolerance of untrained organisms. 

Despite our efforts, this study deserves criticism. Our experimental design does not allow us to state that CV is equal to and significantly correlated with iMLSS in all mouse strains and genders (our findings only refer to healthy male C57BL/6J mice). We also lack time-course analyses on the physiological and molecular responses of mice since the demeanor of MCT1 and MCT4 after an acute exercise session until exhaustion is still uncertain [39]. The construction of an experimental design could make it possible to analyze not only the MCT1 and MCT4 protein content but also several biomarkers and metabolites at different recovery times, which would help to better understand the individual variation in time to exhaustion during maximal aerobic capacity effort. Despite these limitations, our results validate CV as a measure of maximal aerobic capacity with greater consistency and reliability, a significant outcome if we consider its non-invasive character. Therefore, our findings provide new insights and applications in medicine, biology, and exercise physiology, offering tools to many researchers aiming to understand aerobic adaptations in mice exposed to several interventions.

Finally, the present study can contribute to the scientific discussion and practical aspects. The validation of protocols to determine the exercise intensity is undoubtedly important for a better exercise prescription for all the experimental models. Additionally, it is essential to assess the aerobic capacity of individuals since physical exercise is used not only in studies related to performance [88,89] but also in the treatment of diseases [90,91] and aging [92]. Areas such as biology, medicine, and sports science can benefit from this non-invasive protocol to control the training intensity of animals. The efficacy of CV in estimating the aerobic capacity of running mice provides a tool for researchers aiming to better understand training adaptations, especially with regard to aerobic metabolism. In addition, to some extent, our results can not only contribute to rodent studies but also offer new insights and perspectives to those interested in evaluations of physical fitness, including researchers, coaches, and athletes, in an attempt to improve their training routines. Indeed, our data on animals submitted to exercise until exhaustion are very interesting, as they demonstrate exercise-mediated physiological responses that could help exercise physiologists expand their knowledge of the physiological determinants of human performance limits. The transference of knowledge gained from animals to humans seems coherent in a translational way since, despite some obvious differences, rodents and humans present many similar physiological responses when subjected to physical exercise [4,5,93]. By taking as legitimate the translatability of results from the animal model to humans, it is necessary to encourage the use of exercise models in animals to explore complex physiological pathways that are extremely difficult to access in humans. 

## 4. Materials and Methods

### 4.1. Ethics

The experiments were conducted according to the current Brazilian legislation. The Brazilian College of Animal Experimentation standards were rigorously followed, and all procedures were previously approved by the Ethics Committee on the Use of Animals (CEUA) of the University of Campinas (UNICAMP) under protocol #4940-1/2018.

### 4.2. Animals and Laboratory Conditions

A total of 30 male C57BL/6J mice (n = 30) divided into two groups (n = 15 per group) were investigated in this study. The sample size was determined with an a priori power analysis using G*power software (G*Power version 3.1.9.7 Uiversität Kiel, Germany), which calculated a sample size of 15 mice per group based on 5% significance level with 95% power, assuming an effect size of 0.62.

The animals were housed (15 per cage) in polypropylene cages (length: 40 cm; width: 33 cm; height: 16 cm; usable area: 1320 cm^2^) kept on Alesco Classic ventilated shelves (Monte Mor, SP—Brazil). During the experiment, they were kept at a temperature of 22 ± 2 °C and relative humidity of 45–55% under a 12/12 h light/dark cycle (lights remained on from 6 am to 6 pm). The mice were fed balanced Labina-Purina^®^ rodent chow (Vevey, Switzerland) and provided with water ad libitum. Noise in the vivarium remained below 85 decibels. All procedures were performed between 6 pm and 9 pm under special lighting, considering that according to evidence, rodents perform better in this period than in daytime [54]. Since rodents are photosensitive and perceive less light above 560 nm [94], the lamps in the vivarium were fixed inside reflectors surrounded by a red filter (ROSCO^®^, mod.#FIRE19 -São Paulo, SP - Brazil) to block spectral energy below 600 nm.

### 4.3. Experimental Design

Male mice weighing 29.5 ± 2.8 g and aged 170 days were initially exposed to a brief adaptation to treadmill running consisting of 3 days. Afterwards, the rodents were randomly divided into two groups, according to our experimental design: control (C, n = 15), maintained in the same environmental conditions, without any exercise effort and euthanized at rest, and exercised (E, n = 15), submitted to aerobic capacity determination protocols and running exercise until exhaustion before euthanasia.

**Figure 7:**
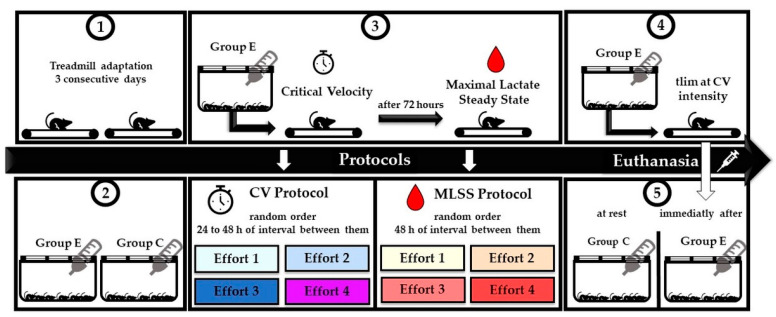
Experimental for the two groups studied: control (C, n = 15) and exercised mice (E, n = 15) (Panel 2). After adaption to treadmill running (panel 1), group E was subjected to the CV protocol, comprised of four efforts performed at intervals of 24 to 48 h at different intensities (predictive CV trials) to obtain each time to exhaustion value (tlim) (Panel 3). The animals were subsequently submitted to the MLSS protocol (Panel 3), characterized by four continuous efforts with blood sample collection performed at intervals of 48 h. Finally, group E was subjected to treadmill running until exhaustion at CV intensity (Panel 4), immediately followed by euthanasia (Panel 5). During the same period, the control group performed no efforts and was euthanized at rest (Panel 5).

Interventions were carried out in group E for about 20 days with the application of the CV and MLSS protocols (both through randomized efforts at different intensities). The mice were submitted to the CV protocol, which consisted of 4 efforts performed at intervals of 24 to 48 h at individual intensities. After 72 h, the rodents were subjected to the MLSS protocol, which was comprised of three or four continuous efforts performed at intervals of 48 h. After 96 h, the animals in group E exercised until exhaustion at CV intensity, being euthanized immediately after effort. Biological materials were collected to determine serum responses, glycogen stores, and the protein content of MCT1 and MCT4 in the skeletal muscle. The experimental design used is summarized in Figure 7. The entire experiment was carried out by highly trained professionals.

### 4.4. Critical Velocity Protocol and Mathematical Models 

**Figure 8:**
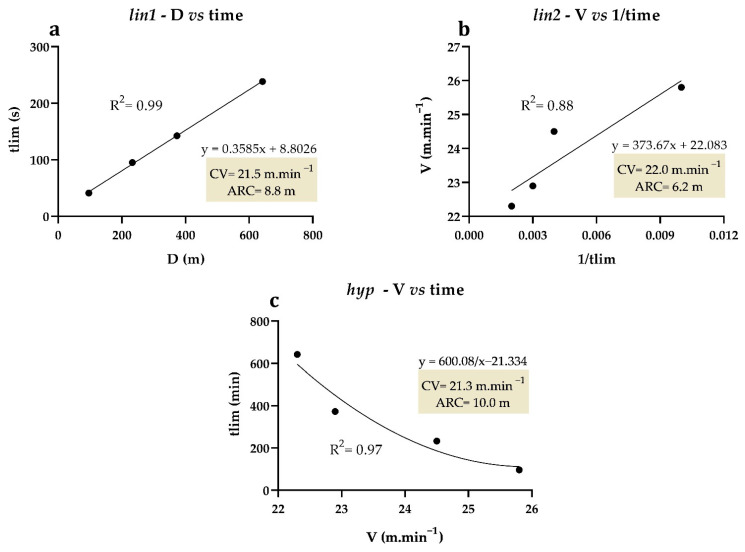
Example taken from an animal for *lin1* (**a**) based on the linear relationship between distance traveled (D) and time, *lin2* (**b**) based on the linear relationship between velocity (V) and the inverse of time (1/time), and *hyp* (**c**) based on the relationship between time and velocity.

The animals were subjected to four continuous running efforts (CV trials) performed at intervals of 24 to 48 h. The exercise intensity in each effort was individually chosen so that exhaustion occurred within 1 to 15 min, as suggested by the adopted model [11,30]. Before testing, each mouse performed a warm-up exercise at 8 m.min^−1^ for 5 min. The tlim value was recorded, and exhaustion was considered the moment when the rodents were unable to correctly run on the treadmill. The tests were conducted without any electrical stimuli. The CV and anaerobic running capacity (ARC) were obtained by three mathematical models (Figure 8), in which these variables were considered the aerobic and anaerobic running parameters, respectively. In the first linear model (*lin1*), the CV and ARC were estimated from the slope of the linear regression line (angular coefficient) and the y-intercept (linear coefficient), respectively, according to the equation D = CV × time + ARC (Figure 8a). In the second linear model (*lin2*), these parameters were obtained by the y-intercept and the slope of the linear regression line between intensity and the inverse of tlim (1/tlim), following the equation V = ARC × 1/time + CV (Figure 8b). Lastly, in the hyperbolic model (*hyp*), the CV and ARC were considered asymptote and curvature constants of the regression models, respectively, according to the equation time = ARC/V − CV (Figure 8c).

### 4.5. Maximal Lactate Steady State Protocol in Treadmill Running

After CV and ARC determination, the mice were submitted to an MLSS protocol consisting of three to four running efforts performed at intervals of 48 h. Each effort lasted 25 min (or until exhaustion) at constant intensities. The average intensity of efforts 1, 2, 3, and 4 were 19.7, 20.7, 21.6, and 23.2 m.min^−1^ [95]. The velocity sequences were randomly distributed, and the intensities were never applied twice to the same rodent. Intensities according to the CV protocol were also useful for MLSS. Blood samples (15 μL) were extracted from the distal end of the animals’ tail at rest and every five minutes of exercise (5, 10, 15, 20, and 25 min). Collections were carried out quickly in less than 30 s. Blood was collected using heparinized capillaries and deposited in 1.5 mL microtubes with 50 μL of 1% sodium fluoride. The lactate concentrations were determined by a lactate analyzer (YSI 2300-STAT-Plus™, Yellow Springs, OH, USA). The lactatemia level during the test was considered stable when no difference above 1 mM was observed between 10 and 25 min of exercise, and the maximal running intensity equivalent to blood lactate concentration was considered the iMLSS [25].

### 4.6. Acute Running until Exhaustion at Individual CV Intensity 

After the application of CV and MLSS protocols, the mice were submitted to continuous exercise at individual CV intensities (obtained by the mathematical model with the best determination coefficients). The time to exhaustion in this running effort was also recorded. The animals were immediately euthanized after exercise in order to evaluate their acute responses to exertion at these intensities. In total, 15 animals of the same age and under similar maintenance conditions were euthanized at the same time of day to allow comparative analyses with the responses at rest. 

### 4.7. Euthanasia and Collection of Biological Material for Analysis

Chemical euthanasia was performed by intraperitoneally administering sodium thiopental at a dose three times higher than that used for anesthesia. After completely losing their reflexes, the mice were subjected to thoracotomy, followed by cardiac puncture for blood removal [77]. The blood samples were centrifuged and stored in serum aliquots [30]. For Western blot assays, the soleus muscles were removed, frozen in liquid nitrogen, and stored at −80 °C (Ultra-Low Temperature Freezer MDF-C8V1, ^©^PHC Corporation of North America, Wood Dale, IL, USA). Liver, heart, kidney, and three muscle portions (vastus lateralis, gastrocnemius, and gluteus maximus) were dissected for tissue glycogen analysis [96]. Finally, visceral (epididymal and retroperitoneal) fat deposits were removed and weighed. 

### 4.8. Biochemical Analyses

Serum samples were used to determine glucose, cholesterol, triacylglycerol, albumin, and urea concentrations. The standards of the commercial kit manufacturer (LaborLab, Guarulhos, SP, Brazil) were followed during the analyses. Tissue glycogen stores were determined by the method described by Dubois et al. [96], which consisted of dissolving liver, cardiac, renal, and muscle tissues in 30% KOH. Then, glycogen was precipitated via sodium sulfate and ethanol (70%) treatment. After a reaction using phenol and sulfuric acid, the glycogen concentrations were colorimetrically measured by absorbance at 490 nm.

### 4.9. Western Blot 

The removed tissues were homogenized in 112 μL of RIPA buffer with the following components: 1% Tris HCl (50 mM), NaCl (150 mM), EDTA (1 mM), IGEPAL^®^ CA-630 (1%), Deoxycholate (0.5%), SDS (0.1%), 1% protease (Protease and Phosphatase Inhibitor Cocktail, cat# P8340, Sigma-Aldrich^®^), and phosphatase inhibitors (Phosphatase II Inhibitor Cocktail Set, cat# US1524625-1SET, Calbiochem^®^, San Diego, CA, USA). After homogenization, a sonicator (Q55 Sonicator, Qsonica®, Newtown, CT, USA)—whose tubes remained immersed in ice—was used for 3 s (60%), followed by refrigerated (4 °C) centrifugation for 10 min at 12,000 rpm (5424 R Centrifuge, Eppendorf AG, Hamburg, Germany). The samples (40 μg of total protein by Bradford method) were mixed with LDS buffer (Lithium dodecyl sulfate and Mercaptoethanol-1%). After loading the wells, electrophoresis was performed using specific equipment (PowerPac 300 Electrophoresis Power Supply—Bio-Rad, São Paulo, SP, Brazil) at 130 V for approximately 60 min. An iBlot™ 2 Gel Transfer Device (20 V for 7 min cat# IB21001, Waltham, MA, USA) was used to transfer proteins (PVDF) to the membrane (Invitrogen™, iBlot™ 2 Transfer Stacks cat# IB# IB24002, Waltham, MA, USA). The membranes were then subjected to fluorescence staining (RevertTM total protein stain, cat # 926-11010) and scanned using the 700 nm channel of a Li-COR Odyssey Fc imaging system. Blocking was performed in 1% milk (skimmed milk powder, cat# 9999, Cell Signaling Technology®, Danvers, MA, USA) in PBS (10 mL of PBS, 0.1 g of milk-NFDM) for 1 h and the membranes were immediately incubated with 5% milk in PBS-Tween for 1 h at room temperature (for MCT4 antibody, cat #BS-2698R, Bioss, Woburn, MA, USA) or overnight at 4 °C (for MCT1, cat # BS-10249R, Bioss, Woburn, MA, USA). Antibody binding was detected by preabsorbed goat anti-rabbit IgG H&L (cat. #ab216773) (IRDye 800CW) at a dilution of 1:20,000 for 1 h at room temperature in the dark. Fluorescence was detected at 800 nm using the same imaging system (Odyssey Fc, LI-COR Biosciences, Lincoln, NE, USA). Protein expression was normalized by dividing the antibody signal by the lane normalization factor [31].

### 4.10. Statistical Analysis

Statistical analyses were performed using Statistica 7.0 software (Statsoft, 2300 Tulsa, OK, USA). Data normality and homogeneity were tested by the Shapiro–Wilk and Levene tests, respectively. The results are expressed as mean ± standard error of the mean (SEM). One-way ANOVA was used to compare tlim obtained in the four predictive efforts of the CV protocol, while analysis of variance (ANOVA) was employed to compare blood lactate concentrations in the four MLSS efforts. The coefficients of determination (R^2^) were calculated to adjust quality indices during mathematical model analyses. As proposed by Copp et al. [28] and Gobatto et al. [97], comparisons between the estimates obtained by mathematical models (in our case, *lin1, lin2*, and *hyp*) were made using ANOVA repeated measurements. The Newman–Keuls post hoc test was adopted when appropriate. The paired sample *t*-test was used to compare the iMLSS and CV in each model. The correlations between iMLSS and CV derived from the mathematical models used were investigated by Pearson’s product–moment and Bland–Altman agreement analysis. Exercised (E)—euthanized after acute running until exhaustion at CV—and control animals (C)—euthanized at rest—were compared via *t*-test for independent samples means. The effect size (ES) was calculated for each paired comparison, providing an estimate of the magnitude of the difference between the groups. The ES was considered small, medium, and large when ES ≤ 0.49, ES = 0.5 to 0.79, and ES ≥ 0.8, respectively [98]. In all cases, a significance level of *p* ≤ 0.05 was adopted.

## 5. Conclusions

In summary, our data reinforced that the CV in running C57BL/6J mice can effectively help estimate their maximum aerobic capacity since there was no difference between the CV intensity obtained by the three mathematical models used and iMLSS, considered the gold standard protocol. Furthermore, although CV was estimated using a non-invasive method, the results in running mice were positively and significantly correlated with iMLSS, an invasive method. During exercise until exhaustion at CV intensity, it was possible to observe an important inter-individual variation in time to exhaustion and the use of energy substrates by mice (especially evidenced by the degradation of hepatic and gluteal glycogen stores), confirming the aerobic performance demonstrated by CV exercises in previous studies. Finally, no difference was found in MCT1 and MCT4 content between the exercised group and the control (rest), at least in the tissue extracted immediately after the exhaustive running effort. The content of MCTs did not explain the inter-individual variation in time to exhaustion at CV intensity since no significant correlations were found between these variables. In further investigations, we suggest the time-course analysis of these proteins in order to deepen the understanding of aerobic exercise tolerance.

## Figures and Tables

**Table 1:** Critical velocity (CV), anaerobic running capacity (ARC), and the coefficient of determination (R^2^) obtained by three mathematical models.

	*lin1*	*lin2*	*hyp*	Repeated Measures ANOVA
CV (m.min^−1^)	21.0 ± 0.9	21.3 ± 0.9	20.6 ± 0.9 _a,b_	F = 8.85, *p* = 0.001
ARC (m)	6.8 ± 1.1	5.6 ± 0,9	9.5 ± 1.6 _a,b_	F = 5.43, *p* = 0.010
R^2^	0.99 ± 0.00	0.71 ± 0.06 _a_	0.85 ± 0.05 _a,b_	F = 13.27, *p* < 0.001

Data (n = 15) are expressed as mean and standard error of the mean. *lin1* = linear model distance vs. tlim; *lin2* = linear model velocity vs. 1/tlim; *hyp* = hyperbolic model; statistical analysis (repeated measures ANOVA): _a,b_: significant differences (*p* < 0.05) in relation to *lin1* and *lin2*, respectively.

**Table 2:** Physiological variables and glycogen stores for the control and exercised groups.

	Control	Exercised	Statistical Analysis
Body composition parameters			
Retroperitoneal fat (%mc)	0.36 ± 0.0	0.36 ± 0.0	t = 0.09; *p* = 0.928; ES = 0.03
Epididymal fat (%mc)	1.5 ± 0.1	1.4 ± 0.1	t = 1.36; *p* = 0.184; ES = 0.50
Serum parameters			
Glucose (mg/dL)	149.7 ± 4.6	131.8 ± 15.0	t = 1.20; *p* = 0.238; ES = 0.48
Cholesterol (mg/dL)	120.0 ± 2.4	125.7 ± 5.9	t = −0.92; *p* = 0.362; ES = 0.36
Triacylglycerol (mg/dL)	53.2 ± 5.8	60.5 ± 10.1	t = −0.64; *p* = 0.527; ES = 0.24
Albumin (g/dL)	3.7 ± 0.1	3.6 ± 0.1	t = 0.23; *p* = 0.812; ES = 0.09
Urea (mg/dL)	98.7 ± 8.3	58.9 ± 7.0 *	t = 3.30; *p* = 0.002; ES = 1.30
Tissue glycogen (ug/100 mg tissue)			
Vastus lateralis	4.8 ± 1.1	5.4 ± 1.0	t = 0.38; *p* = 0.700; ES = 0.15
Gastrocnemius muscle	6.9 ± 0.6	6.6 ± 0.8	t = 0.24; *p* = 0.809; ES = 0.09
Gluteus	10.8 ± 1.4	7.3 ± 0.6 *	t = 2.12; *p* = 0.043; ES = 0.87
Heart	9.2 ± 1.6	6.1 ± 1.0	t = 1.53; *p* = 0.137; ES = 0.59
Kidney	25.0 ± 8.1	12.1 ± 3.6	t = 1.37; *p* = 0.180; ES = 0.55
Liver	46.9 ± 5.4	12.4 ± 1.4 *	t = 5.80; *p* = 0.00; ES = 2.54

* Different from the control group (*p* ≤ 0.05). %mc = relative to body mass.

## Data Availability

Not applicable.

## References

[1] Ghosh S., Golbidi S., Werner I., Verchere B.C., Laher I. (2010). Selecting exercise regimens and strains to modify obesity and diabetes in rodents: An overview. Clin. Sci..

[2] Dawson C.A., Horvath S.M. (1970). Swimming in small laboratory animals. Med. Sci. Sports. Exerc..

[3] Bilu C., Einat H., Kronfeld-Schor N. (2016). Utilization of diurnal rodents in the research of depression. Drug. Dev. Res..

[4] Goutianos G., Tzioura A., Kyparos A., Paschalis V., Margaritelis N.V., Veskoukis A.S., Zafeiridis A., Dipla K., Nikolaidis M.G., Vrabas I.S. (2015). The rat adequately reflects human responses to exercise in blood biochemical profile: A comparative study. Physiol. Rep..

[5] Veskoukis A.S., Goutianos G., Paschalis V., Margaritelis N.V., Tzioura A., Dipla K., Zafeiridis A., Vrabas I.S., Kyparos A., Nikolaidis M.G. (2016). The rat closely mimics oxidative stress and inflammation in humans after exercise but not after exercise combined with vitamin C administration. Eur. J. Appl. Physiol..

[6] Wisløff U., Helgerud J., Kemi O.J., Ellingsen Ø. (2001). Intensity-controlled treadmill running in rats: Vo2 max and cardiac hypertrophy. Am. J. Physiol. Heart Circ. Physiol..

[7] Høydal M.A., Wisløff U., Kemi O.J., Ellingsen O. (2007). Running speed and maximal oxygen uptake in rats and mice: Practical implications for exercise training. Eur. J. Prev. Cardiol..

[8] Booth F.W., Laye M.J., Spangenburg E.E. (2010). Gold standards for scientists who are conducting animal-based exercise studies. J. Appl. Physiol..

[9] Araujo G.G., Gobatto C.A., Manchado-Gobatto F.B., Teixeira L.F., Dos Reis I.G., Caperuto L.C., Papoti M., Bordin S., Cavaglieri C.R., Verlengia R. (2015). MCT1 and MCT4 kinetic of mRNA expression in different tissues after aerobic exercise at maximal lactate steady state workload. Physiol. Res..

[10] Gobatto C.A., Manchado-Gobatto F.B., Carneiro L.G., de Araujo G.G., dos Reis I.G.M. (2009). Maximal lactate steady state for aerobic evaluation of swimming mice. Comp. Exerc. Physiol..

[11] Manchado-Gobatto F.B., Gobatto C.A., Contarteze R.L., Papoti M., Araujo G.G., Mello M.A.R. (2010). Determination of critical velocity and anaerobic capacity of running rats. J. Exerc. Physiol. Online..

[12] Billat V.L., Sirvent P., Py G., Koralsztein J.P., Mercier J. (2003). The concept of maximal lactate steady state: A bridge between biochemistry, physiology and sport science. Sports Med..

[13] Philp A., Macdonald A.L., Carter H., Watt P.W., Pringle J.S. (2008). Maximal lactate steady state as a training stimulus. Int. J. Sports Med..

[14] Beneke R., Hütler M., Leithäuser R.M. (2000). Maximal lactate-steady-state independent of performance. Med. Sci. Sports Exerc..

[15] Jones A.M., Burnley M., Black M.I., Poole D.C., Vanhatalo A. (2019). The maximal metabolic steady state: Redefining the ‘gold standard’. Physiol. Rep..

[16] Beneke R., Leithäuser R.M., Ochentel O. (2011). Blood lactate diagnostics in exercise testing and training. Int. J. Sports Physiol. Perform..

[17] Dekerle J., Baron B., Dupont L., Vanvelcenaher J., Pelayo P. (2003). Maximal lactate steady state, respiratory compensation threshold and critical power. Eur. J. Appl. Physiol..

[18] Wakayoshi K., Ikuta K., Yoshida T., Udo M., Moritani T., Mutoh Y., Miyashita M. (1992). Determination and validity of critical velocity as an index of swimming performance in the competitive swimmer. Eur. J. Appl. Physiol. Occup. Physiol..

[19] Poole D.C. (2009). Resolving the determinants of high-intensity exercise performance. Exp. Physiol..

[20] Vanhatalo A., Jones A.M., Burnley M. (2011). Application of critical power in sport. Int. J. Sports Physiol. Perform..

[21] Morton R.H. (2006). The critical power and related whole-body bioenergetic models. Eur. J. Appl. Physiol..

[22] Gaesser G.A., Poole D.C. (1996). The slow component of oxygen uptake kinetics in humans. Exerc. Sport Sci. Rev..

[23] Fukuda D.H., Smith A.E., Kendall K.L., Dwyer T.R., Kerksick C.M., Beck T.W., Cramer J.T., Stout J.R. (2010). The effects of creatine loading and gender on anaerobic running capacity. J. Strength Cond. Res..

[24] Nakamura F.Y., Pereira G., Hill D.W., Berthoin S., Kokubun E. (2008). There is no anaerobic work capacity replenishment at critical power intensity: An indirect evidence. Sci. Sports..

[25] Billat V.L., Mouisel E., Roblot N., Melki J. (2005). Inter- and intrastrain variation in mouse critical running speed. J. Appl. Physiol..

[26] Ferguson S.K., Redinius K., Yalamanoglu A., Harral J.W., Hyen Baek J., Pak D., Loomis Z., Hassell D., Eigenberger P., Nozik-Grayck E. (2019). Effects of living at moderate altitude on pulmonary vascular function and exercise capacity in mice with sickle cell anaemia. J. Physiol..

[27] Mille-Hamard L., Breuneval C., Rousseau A.S., Grimaldi P., Billat V.L. (2015). Transcriptional modulation of mitochondria biogenesis pathway at and above critical speed in mice. Mol. Cell. Biochem..

[28] Copp S.W., Hirai D.M., Musch T.I., Poole D.C. (2010). Critical speed in the rat: Implications for hindlimb muscle blood flow distribution and fibre recruitment. J. Physiol..

[29] Manchado-Gobatto F.B., de Araujo G.G., Ribeiro C., Araújo M.B., de Alencar Mota C.S., Gobatto C.A., de Mello M.A.R. (2012). Effects of light-dark cycle manipulation on critical velocity and anaerobic running capacity in Wistar rats. Comp. Exerc. Physiol..

[30] Scariot P.P.M., Manchado-Gobatto F.B., Prolla T.A., Masselli Dos Reis I.G., Gobatto C.A. (2019). Housing conditions modulate spontaneous physical activity, feeding behavior, aerobic running capacity and adiposity in C57BL/6J mice. Horm. Behav..

[31] Scariot P.P.M., Manchado-Gobatto F.B., Beck W.R., Papoti M., Van Ginkel P.R., Gobatto C.A. (2022). Monocarboxylate transporters (MCTs) in skeletal muscle and hypothalamus of less or more physically active mice exposed to aerobic training. Life Sci..

[32] Monod H., Scherrer J. (1965). The work capacity of a synergic muscular group. Ergonomics.

[33] Burnley M., Jones A.M. (2007). Oxygen uptake kinetics as a determinant of sports performance. Eur. J. Sport. Sci..

[34] Hargreaves M., Spriet L.L. (2020). Skeletal muscle energy metabolism during exercise. Nat. Metab..

[35] Pérez de Heredia F., Wood I.S., Trayhurn P. (2010). Hypoxia stimulates lactate release and modulates monocarboxylate transporter (MCT1, MCT2, and MCT4) expression in human adipocytes. Pflugers Arch..

[36] Rocha S. (2007). Gene regulation under low oxygen: Holding your breath for transcription. Trends Biochem. Sci..

[37] Lin J., Wu H., Tarr P.T., Zhang C., Wu Z., Boss O., Michael L.F., Puigserver P., Isotani E., Olson E.N. (2002). Transcriptional co-activator PGC-1α drives the formation of slow-twitch muscle fibres. Nat. Metab..

[38] Hon W., Wilson M.I., Harlos K., Claridge T.D., Schofield C.J., Pugh C.W., Maxwell P.H., Ratcliffe P., Stuart D.I., Jones E.Y. (2002). Structural basis for the recognition of hydroxyproline in HIF-1α by pVHL. Nat. Metab..

[39] Nikooie R., Rajabi H., Gharakhanlu R., Atabi F., Omidfar K., Aveseh M., Larijani B. (2013). Exercise-induced changes of MCT1 in cardiac and skeletal muscles of diabetic rats induced by high-fat diet and STZ. J. Physiol. Biochem..

[40] Juel C., Halestrap A.P. (1999). Lactate transport in skeletal muscle—Role and regulation of the monocarboxylate transporter. J. Physiol..

[41] Petersen C., Nielsen M.D., Andersen E.S., Basse A.L., Isidor M.S., Markussen L.K., Viuff B.M., Lambert I.H., Hansen J.B., Pedersen S.F. (2017). MCT1 and MCT4 expression and lactate flux activity increase during white and brown adipogenesis and impact adipocyte metabolism. Sci. Rep..

[42] Halestrap A.P., Price N.T. (1999). The proton-linked monocarboxylate transporter (MCT) family: Structure, function and regulation. Biochem. J..

[43] Brooks G. (2002). Lactate shuttles in nature. Biochem. Soc. Trans..

[44] Poole D.C., Copp S.W., Colburn T.D., Craig J.C., Allen D.L., Sturek M., O’Leary D.S., Zucker I.H., Musch T.I. (2020). Guidelines for animal exercise and training protocols for cardiovascular studies. Am. J. Physiol. Heart Circ. Physiol..

[45] Copp S.W., Davis R.T., Poole D.C., Musch T.I. (2009). Reproducibility of endurance capacity and VO2peak in male Sprague-Dawley rats. J. Appl. Physiol..

[46] Jenkins D., Kretek K., Bishop D. (1998). The duration of predicting trials influences time to fatigue at critical power. J. Sci. Med. Sport..

[47] Bull A.J., Housh T.J., Johnson G.O., Rana S.R. (2008). Physiological responses at five estimates of critical velocity. Eur. J. Appl. Physiol..

[48] Housh T.J., Cramer J.T., Bull A.J., Johnson G.O., Housh D.J. (2001). The effect of mathematical modeling on critical velocity. Eur. J. Appl. Physiol..

[49] Gaesser G.A., Carnevale T.J., Garfinkel A., Walter D.O., Womack C.J. (1995). Estimation of critical power with nonlinear and linear models. Med. Sci. Sports Exerc..

[50] de Lucas R.D., de Souza K.M., Costa V.P., Grossl T., Guglielmo L.G.A. (2013). Time to exhaustion at and above critical power in trained cyclists: The relationship between heavy and severe intensity domains. Sci. Sports..

[51] Brickley G., Doust J., Williams C. (2002). Physiological responses during exercise to exhaustion at critical power. Eur. J. Appl. Physiol..

[52] Jenkins D.G., Quigley B.M. (1990). Blood lactate in trained cyclists during cycle ergometry at critical power. Eur. J. Appl. Physiol..

[53] Dotan R. (2022). A critical review of critical power. Eur. J. Appl. Physiol..

[54] Beck W.R., Scariot P.P., Gobatto C.A. (2016). Melatonin is an ergogenic aid for exhaustive aerobic exercise only during the wakefulness period. Int. J. Sports Med..

[55] Tirone T.A., Brunicardi F.C. (2001). Overview of glucose regulation. World J. Surg..

[56] Greenberg C.C., Jurczak M.J., Danos A.M., Brady M.J. (2006). Glycogen branches out: New perspectives on the role of glycogen metabolism in the integration of metabolic pathways. Am. J. Physiol. Endocrinol. Metab..

[57] Holloszy J.O., Kohrt W.M. (1996). Regulation of carbohydrate and fat metabolism during and after exercise. Annu. Rev. Nutr..

[58] Romijn J.A., Coyle E.F., Sidossis L.S., Gastaldelli A., Horowitz J.F., Endert E., Wolfe R.R. (1993). Regulation of endogenous fat and carbohydrate metabolism in relation to exercise intensity and duration. Am. J. Physiol. Endocrinol. Metab..

[59] Jensen T.E., Richter E.A. (2012). Regulation of glucose and glycogen metabolism during and after exercise. J. Physiol..

[60] Mergenthaler P., Lindauer U., Dienel G.A., Meisel A. (2013). Sugar for the brain: The role of glucose in physiological and pathological brain function. Trends Neurosci..

[61] Beck W.F., De Araujo G.G., Menezes Scariot P.P., Masselli dos Reis I.G., Gobatto C.A. (2014). Time to exhaustion at anaerobic threshold in swimming rats: Metabolic investigation. Bratisl. Lek. Listy..

[62] Walther T., Wessel N., Kang N., Sander A., Tschöpe C., Malberg H., Bader M., Voss A. (2000). Altered heart rate and blood pressure variability in mice lacking the Mas protooncogene. Braz. J. Med. Biol..

[63] Wickman K., Nemec J., Gendler S.J., Clapham D.E. (1998). Abnormal heart rate regulation in GIRK4 knockout mice. Neuron..

[64] Lakin R., Guzman C., Izaddoustdar F., Polidovitch N., Goodman J.M., Backx P.H. (2018). Changes in heart rate and its regulation by the autonomic nervous system do not differ between forced and voluntary exercise in mice. Front. Physiol..

[65] De Angelis K., Wichi R.B., Jesus W.R., Moreira E.D., Morris M., Krieger E.M., Irigoyen M.C. (2004). Exercise training changes autonomic cardiovascular balance in mice. J. Appl. Physiol..

[66] Kaplan M.L., Cheslow Y., Vikstrom K., Malhotra A., Geenen D.L., Nakouzi A., Leinwand L.A., Buttrick P.M. (1994). Cardiac adaptations to chronic exercise in mice. Am. J. Physiol. Endocrinol. Metab..

[67] Salazar J.H. (2014). Overview of urea and creatinine. Lab. Med..

[68] Bellinghieri G., Savica V., Santoro D. (2008). Renal alterations during exercise. J. Ren. Nutr..

[69] Poortmans J.R., Vanderstraeten J. (1994). Kidney function during exercise in healthy and diseased humans. An update. Sports Med..

[70] Sokal P., Jastrzębski Z., Jaskulska E., Sokal K., Jastrzębska M., Radzimiński L., Dargiewicz R., Zieliński P. (2013). Differences in blood urea and creatinine concentrations in earthed and unearthed subjects during cycling exercise and recovery. Evid. Based Complement. Altern. Med..

[71] Baker S.K., McCullagh K.J., Bonen A. (1998). Training intensity-dependent and tissue-specific increases in lactate uptake and MCT-1 in heart and muscle. J. Appl. Physiol..

[72] Astorino T., Baker J., Brock S., Dalleck L., Goulet E., Gotshall R., Zhou B. (2015). Lactate and monocarboxylate transporters (MCTS): A review of cellular aspects. J. Exerc. Physiol. Online.

[73] Dimmer K.S., Friedrich B., Lang F., Deitmer J.W., Bröer S. (2000). The low-affinity monocarboxylate transporter MCT4 is adapted to the export of lactate in highly glycolytic cells. Biochem. J..

[74] Bröer S., Schneider H.P., Bröer A., Rahman B., Hamprecht B., Deitmer J.W. (1998). Characterization of the monocarboxylate transporter 1 expressed in Xenopus laevis oocytes by changes in cytosolic pH. Biochem. J..

[75] McCullagh K.J., Poole R.C., Halestrap A.P., O’Brien M., Bonen A. (1996). Role of the lactate transporter (MCT1) in skeletal muscles. Am. J. Physiol. Endocrinol. Metab..

[76] Dubouchaud H., Butterfield G., Wolfel E., Bergman B.C., Brooks G.A. (2000). Endurance training, expression, and physiology of LDH, MCT1, and MCT4 in human skeletal muscle. Am. J. Physiol. Endocrinol. Metab..

[77] Forte L.D.M., Rodrigues N.A., Cordeiro A.V., de Fante T., Simino L.A., Torsoni A.S., Torsoni M.A., Gobatto C.A., Manchado-Gobatto F.B. (2022). Effect of acute swimming exercise at different intensities but equal total load over metabolic and molecular responses in swimming rats. J. Muscle Res. Cell Motil..

[78] Forte L.D.M., Rodrigues N.A., Cordeiro A.V., de Fante T., Simino L.A.P., Torsoni A.S., Torsoni M.A., Gobatto C.A., Manchado-Gobatto F.B. (2020). Periodized versus non-periodized swimming training with equal total training load: Physiological, molecular and performance adaptations in Wistar rats. PLoS ONE.

[79] Thomas C., Bishop D.J., Lambert K., Mercier J., Brooks G.A. (2012). Effects of acute and chronic exercise on sarcolemmal MCT1 and MCT4 contents in human skeletal muscles: Current status. Am. J. Physiol. Regul. Integr. Comp. Physiol..

[80] Thomas C., Perrey S., Lambert K., Hugon G., Mornet D., J.M. (2005). Monocarboxylate transporters, blood lactate removal after supramaximal exercise, and fatigue indexes in humans. J. Appl. Physiol..

[81] Kitaoka Y., Endo Y., Mukai K., Aida H., Hiraga A., Takemasa T., Hatta H. (2013). Effect of acute exercise on monocarboxylate transporters 1 and 4 in untrained and trained Thoroughbreds. Am. J. Vet. Res..

[82] Liu Y., Beyer A., Aebersold R. (2016). On the Dependency of Cellular Protein Levels on mRNA Abundance. Cell.

[83] Mehra A., Lee K.H., Hatzimanikatis V. (2003). Insights into the relation between mRNA and protein expression patterns: I. Theoretical considerations. Biotechnol. Bioeng..

[84] Coles L., Litt J., Hatta H., Bonen A. (2004). Exercise rapidly increases expression of the monocarboxylate transporters MCT1 and MCT4 in rat muscle. J. Physiol..

[85] Kitaoka Y., Takahashi K., Hatta H. (2022). Inhibition of monocarboxylate transporters (MCT) 1 and 4 reduces exercise capacity in mice. Physiol. Rep..

[86] Harley Y.X., Kohn T.A., St Clair Gibson A., Noakes T.D., Collins M. (2009). Skeletal muscle monocarboxylate transporter content is not different between black and white runners. Eur. J. Appl. Physiol..

[87] Bentley D.J., Roels B., Thomas C., Ives R., Mercier J., Millet G., Cameron-Smith D.J.E.j.o.a.p. (2009). The relationship between monocarboxylate transporters 1 and 4 expression in skeletal muscle and endurance performance in athletes. Eur. J. Appl. Physiol..

[88] Murase T., Haramizu S., Shimotoyodome A., Tokimitsu I., Hase T. (2006). Green tea extract improves running endurance in mice by stimulating lipid utilization during exercise. Am. J. Physiol. Regul. Integr. Comp. Physiol..

[89] Eizenga M.R., Flewwelling L.D., Warrier T., Scott G.R. (2023). Thermal performance curve of endurance running at high temperatures in deer mice. J. Exp. Biol..

[90] Cho J., Lee I., Kim D., Koh Y., Kong J., Lee S., Kang H. (2014). Effect of aerobic exercise training on non-alcoholic fatty liver disease induced by a high fat diet in C57BL/6 mice. J. Exerc. Nutrition. Biochem..

[91] Guo S., Huang Y., Zhang Y., Huang H., Hong S., Liu T. (2020). Impacts of exercise interventions on different diseases and organ functions in mice. J. Sport Health Sci..

[92] Dhillon R.S., Qin Y., Van Ginkel P.R., Fu V.X., Vann J.M., Lawton A.J., Green C.L., Manchado-Gobatto F.B., Gobatto C.A., Lamming D. (2022). SIRT3 deficiency decreases oxidative metabolism capacity but increases lifespan in male mice under caloric restriction. Aging. Cell.

[93] Ayachi M., Niel R., Momken I., Billat V.L., Mille-Hamard L. (2016). Validation of a ramp running protocol for determination of the true VO_2_max in mice. Front. Physiol..

[94] Jacobs G.H., Fenwick J.A., Williams G.A. (2001). Cone-based vision of rats for ultraviolet and visible lights. J. Exp. Biol..

[95] Barros Manchado F.B., Gobatto C.A., Contarteze R.V.L., Papoti M., Mello M.A.R. (2005). Maximal lactate steady state in running rats. J. Appl. Physiol..

[96] Dubois M., Gilles K.A., Hamilton J.K., Rebers P.A., Smith F. (1956). Colorimetric method for determination of sugars and related substances. Anal. Chem..

[97] Gobatto C.A., Scariot P.P.M., Ribeiro L.F.P., Manchado-Gobatto F.B. (2013). Critical load estimation in young swimming rats using hyperbolic and linear models. Comp. Exerc. Physiol..

[98] Cohen J. (2013). Book Statistical Power Analysis for the Behavioral Sciences.

